# Determination of Urinary Gluten Immunogenic Peptides to Assess Adherence to the Gluten-Free Diet: A Randomized, Double-Blind, Controlled Study

**DOI:** 10.14309/ctg.0000000000000411

**Published:** 2021-10-06

**Authors:** Chiara Monachesi, Anil K. Verma, Giulia N. Catassi, Elisa Franceschini, Simona Gatti, Rosaria Gesuita, Elena Lionetti, Carlo Catassi

**Affiliations:** 1 Celiac Disease Research Laboratory, Polytechnic University of Marche, Ancona, Italy;; 2 Division of Pediatrics, DISCO Department, Polytechnic University of Marche, Ancona, Italy;; 3 Center of Epidemiology and Biostatistics, Polytechnic University of Marche, Ancona, Italy;; 4 Mucosal Immunology and Biology Research Center, Division of Pediatric Gastroenterology and Nutrition, Massachusetts General Hospital, Boston, USA.

## Abstract

The adherence to a gluten-free diet (GFD) is a trending topic in the management of celiac disease. The aim of our study was to evaluate the diagnostic performance of urinary gluten immunogenic peptides (GIP) determination to detect gluten contamination of the GFD.

**METHODS:**

In study A, 25 healthy adults on a standard GFD performed 6 gluten challenges (0, 10, 50, 100, 500, and 1,000 mg) with quantification of urinary GIP before (T_0_) and during the following 24 hours. In study B, 12 participants on a gluten contamination elimination diet underwent urinary GIP determination at T_0_ and after challenge with 5 or 10 mg gluten. Urine GIP concentration was determined by an immunochromatographic assay.

**RESULTS:**

In study A, 51 of 150 baseline urine samples were GIP+ on GFD and 7 of 17 were GIP+ after the zero-gluten challenge, whereas only 55 of 81 were GIP+ after the 10–1,000 mg gluten challenges. There was no significant change in the 24-hour urinary GIP when increasing gluten from 10 to 1,000 mg. In study B, 24 of 24 baseline urine samples were GIP−, whereas 8 of 24 were GIP+ after 5 or 10 mg of gluten.

**DISCUSSION:**

Traces of gluten in the standard GFD may cause positivity of urinary GIP determination, whereas a false negativity is common after a gluten intake of 10–1,000 mg. Owing to the impossibility of standardizing the test in normal conditions, it seems unlikely that urinary GIP determination may represent a reliable tool to assess the compliance to the GFD of patients with celiac disease or other gluten-related disorders.

## INTRODUCTION

Celiac disease (CD) is a systemic autoimmune disorder triggered by the ingestion of gluten in genetically predisposed individuals (1). It is one of the most frequent lifelong diseases, affecting approximately 1%–2% of the general population worldwide (2). A gluten-free diet (GFD), the only effective treatment of CD, determines clinical, serological, and histological remission and prevents long-term CD complications (3). However, a strict GFD is extremely difficult to maintain. Gluten is indeed a pervasive ingredient that may be used as a protein filler in a huge number of commercial foods (e.g., sausages, soups, soy sauces, and hamburgers) or may contaminate originally gluten-free products in the production chain (4). Unfortunately, even traces of gluten in the diet (≥10 mg/d) are sufficient to cause damage to the celiac small intestinal mucosa when ingested repeatedly (5).

Hence, it is very important to monitor GFD adherence of patients with CD. Dietary interview, clinical symptoms monitoring, CD serology, and small intestinal histology are significant choices; however, they provide only limited and indirect evidence of GFD adherence (6–8). Moreover, these tools are inadequately sensitive to detect the accidental exposure to traces of dietary gluten. Novel qualitative and quantitative immunochromatographic tests have been developed to directly detect recent dietary exposure to gluten by determining the excretion of gluten immunogenic peptides (GIP) in stools or urine (9–11). A growing interest has recently focused on the role of stool/urinary GIP determination in the follow-up of treated patients with CD, and this noninvasive and easy to perform test seems to be the most promising and reliable marker of dietary gluten transgressions (12–22). Inadequate information is available about the relationship between the amount of ingested gluten and the quantity of GIP excreted in urine or stool particularly at a low level of gluten ingestion (as is usually the case in treated patients with CD).

The aim of this study was to assess the diagnostic performance of urinary GIP determination and the dose-response relationship between the amount of ingested gluten and the quantity of GIP recovered in urine, in a group of healthy and qualified volunteers adhering to a GFD and undergoing repeated dietary challenges with increasing amounts of gluten.

## PATIENTS AND METHODS

This study was a randomized, double-blind, controlled study aimed to investigate the relationship between the increasing amount of ingested gluten and the quantity of GIP in urine.

### Participants

This study was conducted on a group of healthy young medical doctors who were all pediatric residents in the Division of Pediatrics at the DISCO Department of the Polytechnic University of Marche, Ancona, Italy. Written informed consent was obtained from each participant. The exclusion criteria were any chronic or acute disease, pregnancy or lactation, chronic intake of medications or supplements, or refusal/withdrawal of written informed consent. Before the study, serum immunoglobulin A (IgA) class anti-transglutaminase antibody was determined in all participants to exclude active CD.

### Study design

Each participant underwent a random sequence of single-dose gluten challenges, collection of all urine excreted during the following 24 hours, and quantification of urinary GIP. Recent data showed that urinary GIP are undetectable after 16–34 hours from the complete removal of gluten from the diet (11). Therefore, to guarantee the complete absence of urinary GIP at baseline, a strict GFD was started 3 days (72 hours) before each gluten challenge and continued for 24 hours after the gluten challenge. A urine sample was collected at T_0_ (first morning urine after 3 days of GFD and immediately before the gluten challenge). After the gluten challenge, all urine excreted during the next 24 hours was collected into 2 different sterile containers (1 for the first 9 hours [T_0_-T_9_ collection] and the other for the following 13 hours [T_10_-T_24_ collection]), and the total volumes were measured. Sample timing (T_9_ and T_24_) was based on previous data suggesting that GIP is detected in urine between 3 and 9 hours from gluten reintroduction (11).

**Figure 1.**
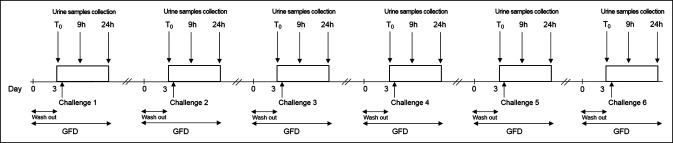
Flow chart of study A. GFD, gluten-free diet; T_0,_ baseline.

This study consisted of 2 parts (A and B), each characterized by a different approach to the GFD and by different gluten doses administered with the challenge. During study A, all participants were instructed to follow 6 bouts of a standard GFD (see Dietary Interventions section). The participants were randomized to a sequence of 6 gluten challenges (0, 10, 50, 100, 500, and 1,000 mg of gluten) (Figure 1). The gluten was administered in capsules prepared by our hospital pharmacy. Each capsule contained a weighed amount of raw gluten.

Study B was deemed necessary after analyzing the results of study A (see Results section). In study B, a subgroup of randomly chosen participants underwent 2 further gluten challenges in a random sequence with either 5 or 10 mg of gluten while performing the gluten contamination elimination diet (GCED) (23). Doses of 5 and 10 mg are tiny amounts of gluten that are still tolerable and may be found in a standard GFD (5).

### Outcome

The primary outcome of this study was the correlation between the amount of ingested gluten and the quantity of GIP excreted in urine during the following 24 hours.

### Ethical clearance

This study was conducted in accordance with the principles of the Helsinki Declaration as revised in Fortaleza 2013 and was approved by the Ethical Committee of the Polytechnic University of Marche, Ancona, Italy (ID #131530). The trial was registered in the clinicaltrials.gov registry (ClinicalTrials.gov ID #NCT04477239).

### Randomization

Randomization was performed using a random sequence generator (Research Randomizer, Version 4.0; https://www.randomizer.org/).

### Dietary interventions

In study A, the GFD (see GFD Protocol, Supplementary Material 1, Supplementary Digital Content 1, http://links.lww.com/CTG/A702) included commercially labeled and certified gluten-free food, that is, items containing less than 20 mg/kg (20 parts per million = ppm) of gluten (e.g., gluten-free bread, pasta, pizza, and flour) ensuring a daily gluten intake of ≤10 mg gluten per day, according to international regulations.

In study B, the GCED was designed to eliminate any possible source of gluten exposure, including the minute gluten traces (<20 ppm) that are allowed in a standard GFD (23). To achieve the elimination of any possible source of gluten in the diet, almost all processed foods, even those foods labeled gluten-free, were removed; only whole, fresh unprocessed foods were allowed. As for cereals, only rice was allowed. The GCED scheme is shown in Supplementary Material 2, Supplementary Digital Content 2, http://links.lww.com/CTG/A703.

All the dietary schemes were administered by a dietitian with expertise in the treatment of CD. All participants were medical doctors with background knowledge of CD and of the GFD. They were further educated about GFD restrictions. Participants were required to report each food/meal consumed during the 3 days of GFD in a food diary. The study was conducted during a period of time characterized by severe restrictions in dining out imposed by the coronavirus disease 2019 pandemic, a circumstance that facilitated the participants' adherence to the dietary regimens of the study.

### Urine sampling and storage

All participants were provided with sterile containers and tubes for urine collection. For each gluten challenge, 3–5 mL urine samples were taken: (i) at baseline (after 3 days of GFD or GCED diet) and after the gluten challenge, (ii) from urine collected between T_0_ and T_9_, and (iii) from urine collected between T_10_ and T_24_. Volunteers were asked to keep the urine container at 4 °C and to record the volume of the T_0_-T_9_ and T_10_-T_24_ urine collections. The 5 mL aliquots were stored at −20 °C until delivered to the laboratory for analysis.

### Quantification of GIP in urine samples

All laboratory tests were performed at the Celiac Disease Research Laboratory, Polytechnic University of Marche, Ancona, Italy. Urine GIP concentration was determined using the rapid immunochromatographic assay based on anti-gliadin 33-mer G12 monoclonal antibodies iVYCHECK GIP Urine test (In Vitro Diagnostics, Biomedal, Spain), according to the manufacturer's instructions. Samples showing a nonquantifiable readout (indicating the presence of 2.2–6.3 ng GIP/mL urine) were approximated to 4 ng/mL in the calculations below.

The urinary GIP excretion was expressed as ng/mL and as ng/24 hours on the total volume of urine collected during the 24 hours after the challenge.

### Determination of serum IgA anti-tissue transglutaminase antibody

IgA anti-tissue transglutaminase antibody assay was performed in our laboratory by a fluorescence enzyme immunoassay ≤30 days before the start of the study (normal values < 7 U/mL).

### Statistical analysis

Sample size was calculated considering a repeated measures analysis of variance model using the expected difference in the mean urinary GIP excretion after gluten challenge and zero-gluten challenge as the primary response variable.

Demographic data are presented as mean and SD or median and interquartile range (first–third quartiles) for the quantitative variables or absolute frequencies and percentages for the qualitative variables. The Shapiro-Wilk test was used to assess the normal distribution of the variables. The Wilcoxon signed-rank test was used to compare continuous variables. The χ^2^ test for trend was used to test the frequency equality of positive results on 6 baseline assessment challenges. Spearman's correlation coefficients and 95% confidence interval (CI) were used to estimate correlation between urinary GIP concentration evaluated at T_0_–T_9_ and T_10_-T_24_. A linear regression model with mixed random effects, which defined the subject as a random factor, was used to estimate the association between urinary GIP recovery in 24 hours (ng/24 hours) and the 6 increasing doses of gluten consumption (mg). In the regression framework, the GIP concentration acted as a dependent variable determined by the doses of gluten transformed on a logarithmic scale. Regression coefficients were estimated by 90% CI. The receiver operating characteristics (ROC) analysis was performed to evaluate sensitivity and specificity of urinary GIP, considering dose 0 mg (zero-gluten challenge) of gluten as the reference dose. The results are showed graphically reporting the observed and the estimated ROC curve with 90% confidence bands. Area under curve (AUC) and 90% CI were also estimated. Statistical analysis was performed using R software (version 4.0.2, 2019; R Core Team, Vienna; Austria), IBM SPSS Statistic v.23.0 (SPSS, Chicago, IL), and Microsoft Excel (v.2010; Microsoft Corp Redmond, Washington, DC).

## RESULTS

### Participants

Forty-five residents were eligible for participation; 25 accepted to participate, and all completed the study between October 2020 and February 2021. There were 21 women (84%) and 4 men (16%), reflecting the higher female prevalence among residents in pediatrics, with a mean age of 31 years (SD 2, age range: 26–33 years). All participants showed a normal result of the serum IgA anti-transglutaminase determination.

### Study A

#### Dietary compliance

Based on the analysis of the 24-hour food diary, no participant reported transgression to the GFD except one who inadvertently violated the protocol by tasting a gluten-containing cake during challenge n.4. The 2 urine samples after this challenge were eliminated from further analysis.

#### Urinary GIP determinations

Overall, 448 urinary samples were analyzed, 150 baseline (6 tests for 25 participants) and 298 after challenge (2 samples—T_0_–T_9_ and T_10_–T_24_—for 149 challenge procedures).

**Figure 2.**
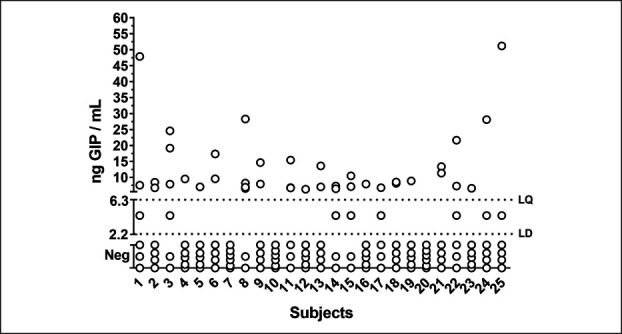
Baseline (T_0_) urinary gluten immunogenic peptides determinations after 3 days of standard gluten-free diet. GIP, gluten immunogenic peptides; LD, limit of detection; LQ, limit of quantification.

Figure 2 shows the results of the baseline urinary GIP determinations. Fifty-one of 150 baseline urine samples (34%) were positive for GIP, 40 (27%) with a quantifiable readout (median 8.21 ng/mL, range 6.30–51.18), and 11 (7%) below the quantification limit. No significant trend in the frequency of GIP+ baseline samples was observed from the first to the sixth challenge (36, 40, 28, 36, 36, and 28, respectively; *P* = 0.579).

As for postchallenge samples, no significant difference was detected in the distribution of urinary GIP concentration between T_0_-T_9_ and T_10_-T_24_ collections for each dose of gluten, and no significant correlation was found (Table 1).

**Table 1.** Comparison of gluten immunogenic peptides concentration (ng/mL) between 0–9 and 10–24 hours of urine collections

Gluten dose (mg)	T_0_–T_9_ collection	T_10_–T_24_ collection	*P*	r (95%CI)
	Median (IQR)	Median (IQR)		
0	0 (0–4)	0 (0–6)	0.831	0.21 (−0.20; 0.56)
10	4 (0–7)	4 (0–14)	0.521	−0.05 (−0.45; 0.36)
50	0 (0–4)	0 (0–7)	0.357	0.26 (−0.15; 0.59)
100	0 (0–0)	4 (0–10)	0.052	−0.04 (−0.43; 0.36)
500	4 (0–7)	4 (0–11)	0.241	0.09 (−0.31; 0.47)
1,000	8 (0–16)	0 (0–7)	0.066	0.04 (−0.37; 0.44)

CI, confidence interval; IQR, interquartile range; r, Spearman correlation coefficient.

*P* value refers to the Wilcoxon signed-rank sum test.

After excluding urine samples collected from GIP+ participants at baseline, there were 7 of the 17 participants (41%) with GIP+ urine samples after the zero-gluten challenge, 4 (24%) of them on both T_0_–T_9_ and T_10_–T_24_ samples, 1 (6%) on T_0_–T_9_ only, and 2 (12%) on T_10_–T_24_ only. After the gluten challenge (10–1,000 mg), 55 of 81 urine samples (68%) showed urinary GIP positivity, 18 of 55 on T_0_–T_9_ only, 15 of 55 on T_10_–T_24_ only, and 22 on both T_0_–T_9_ and T_10_–T_24_ samples. In detail, 15 of the 18 participants (83%) showed T_0_–T_9_ and/or T_10_–T_24_ GIP+ urine samples after challenge with 10 mg, 12 of 20 (60%) with 50 mg, 10 of 19 (53%) with 100 mg, 10 of 13 (77%) with 500 mg, and 8 of 11 (73%) with 1,000 mg of gluten.

#### Dose/response relationship

**Figure 3.**
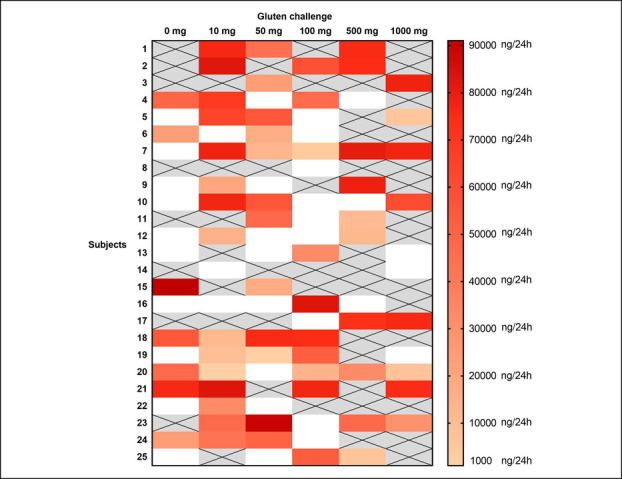
Results of the 24-hour urinary GIP determination after the different gluten challenges in subjects performing the standard gluten-free diet. Crossed gray cells: samples excluded because of a positive T0 result. White cells: GIP-negative samples. GIP, gluten immunogenic peptides.

**Figure 4.**
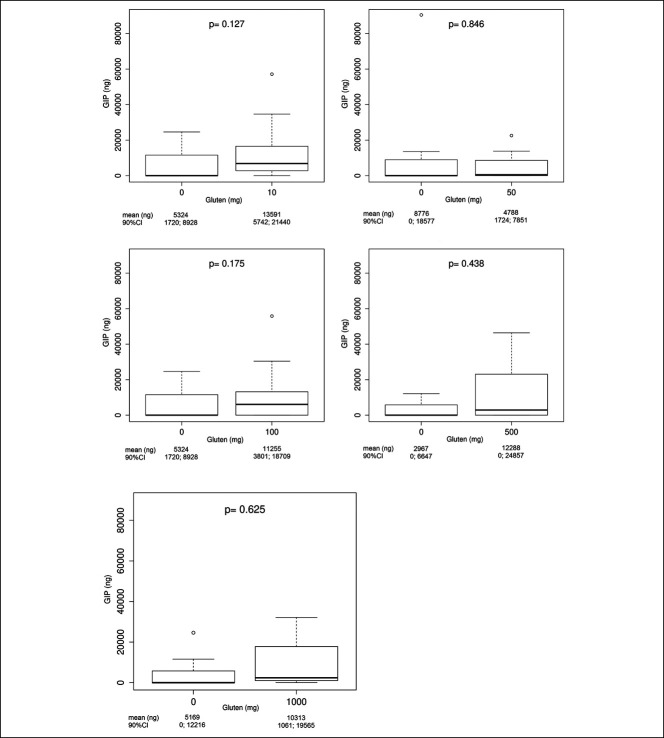
Comparison between the zero-gluten challenge and the gluten challenge urinary GIPs responses at different levels of gluten intake. *P* values refer to the Wilcoxon signed-rank test. GIP, gluten immunogenic peptides.

**Figure 5.**
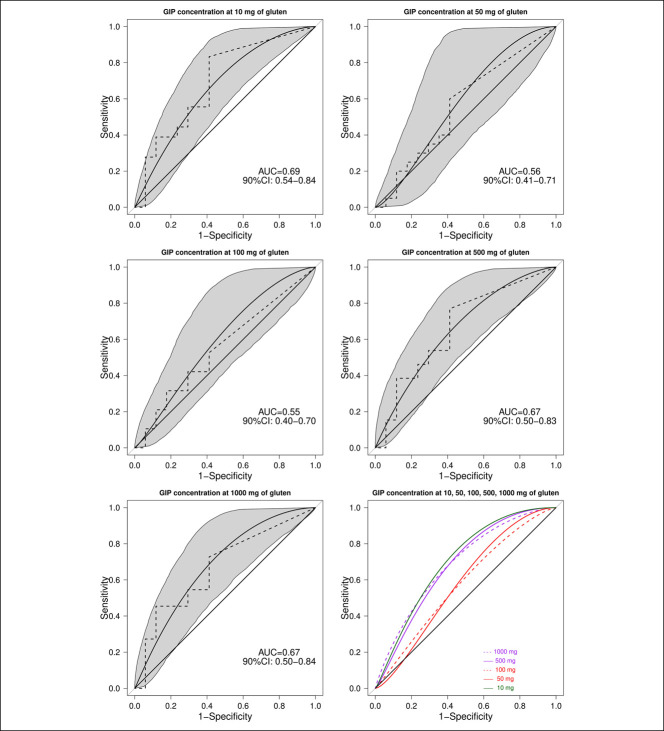
Observed (dotted line) and estimated ROC curves (solid line) with 90% confidence bands (gray area). AUC, area under curve; CI, confidence interval; ROC, receiver operating characteristics.

Figure 3 shows the 24-hour urinary GIP recovery after each challenge procedure, expressed as ng/24 hours. Figure 4 shows the comparison between the zero-gluten challenge and the gluten challenge responses for each gluten level, after exclusion of all samples belonging to the baseline GIP+ subjects. There was no significant difference between the zero-gluten challenge and the gluten challenge response for all doses of gluten. The regression coefficient estimated that the mixed-effect linear model was equal to 96 (95% CI = −518; 709), showing no significant change in urinary GIP content when the gluten dose increased from 0 to 1,000 mg. Figure 5 shows the results of the ROC analysis, considering the zero-gluten dose as reference. Doses 10, 500, and 1,000 mg had AUC values between 0.67 and 0.69, with the lower limits of 90% CIs close to 0.50. Doses 50 and 100 mg showed observed AUC values close to 0.50, with the observed and estimated ROC values very close to the diagonal of the graph.

### Study B

#### Dietary compliance

Based on the analysis of the 24-hour food diary, no participant reported transgression to the GCED.

#### Urinary GIP determinations

**Figure 6.**
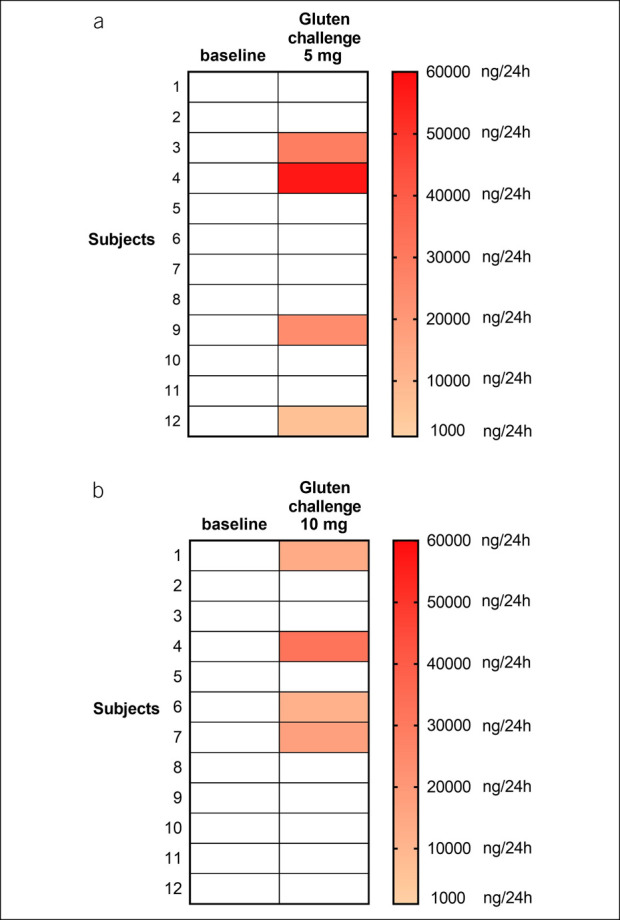
Results of 24-hour urinary GIP determination before and after the 5 mg (**a**) and the 10 mg (**b**) gluten challenge in subjects performing the gluten contamination elimination diet. White cells: GIP-negative samples. GIP, gluten immunogenic peptides.

After 3 days of GCED, baseline urine samples (n = 24) constantly tested negative for GIP (Figures 6 a,b). After the challenge with microdoses of gluten, 8 of 24 (33%) showed GIP positivity. In detail, 3 of 12 participants were GIP positive after taking 5 mg of gluten (2 only on the T_0_–T_9_ sample and 1 on both T_0_–T_9_ and T_10_–T_24_ samples), 3 of 12 were positive after taking 10 mg (2 only on the T_9_ sample and 1 on both T_9_ and T_24_ samples), and 1 was positive at both doses, on both T_0_–T_9_ and T_10_–T_24_ samples after 5 mg and on the T_0_–T_9_ sample after 10 mg of gluten.

## DISCUSSION

In a group of healthy and qualified volunteers undergoing dietary challenges with increasing amounts of gluten, the performance of urinary GIP determination in monitoring the GFD was poor. Indeed, a significant percentage of subjects had a positive GIP determination on a strict GFD (34%) and/or after the zero-gluten challenge (41%). At the same time, a high percentage of subjects had a negative GIP determination after challenges with a significant amount of gluten (up to 1 g).

In the past few years, a growing interest has focused on the assessment of compliance to the GFD, and GIP determination in stools or urine has been the most promising tool (12–22). GIP are fragments of gluten proteins that are reactive to the anti–33-mer G12 monoclonal antibody. A small fraction of ingested gluten peptides is either adsorbed and excreted in urine or excreted in stools, thereby revealing ongoing gluten exposure. Fecal GIP positivity has been found in 16%–30% of treated patients with CD (8,10,12,13). In a systematic review, the GIP assay showed the lowest celiac dietary adherence rate (75%) in children with CD on a GFD as compared with the intestinal biopsy (87%), self-report (81%), structured dietary interview (77%), and CD serological markers (76%), suggesting that this test is more sensitive than other methods of GFD monitoring (6). Healing of the small intestinal mucosa has been associated with the repeated absence of urinary GIP in treated patients with CD (7,21). However, previous studies assumed that the absence or presence of GIP in urine directly reflects absence or presence of contaminating gluten in the GFD, an axiom that has never been investigated in depth. As for the dose/response relationship, the only available data showed that 3/4 and 4/4 out of 4 healthy subjects kept on the GFD had at least 1 positive urinary GIP test after a challenge with 25 or 50 mg of gluten, respectively (11). It should be noted that 25 mg is a tiny amount of gluten, close to the maximum amount that is tolerable in the standard GFD, that is, 10 mg/d (5,24).

Our study evaluated the performance of urinary GIP determination in a randomized, double-blind, controlled gluten challenge trial for the first time. We found that 34% of healthy subjects showed a positive urinary GIP test after a strictly controlled standard GFD for 3 days. After excluding subjects with GIP positivity at baseline, 41% had a positive GIP determination after a zero dose of gluten. At first glance, these disappointing results could be explained by (i) prolonged urinary GIP elimination (≥3 days) after stopping the gluten-containing diet, (ii) overestimation of false positives secondary to testing urine collections instead of random urine samples, and (iii) poor compliance with the GFD. Despite many precautions taken to avoid dietary transgression (the strong motivation of participants and their professional awareness of the GFD requirements, in-depth monitoring of the diet, and impossibility to dine out because of coronavirus disease 2019–related restrictions), we cannot exclude dietary mistakes in participants who followed the GFD for only 72 hours. However, the results of study B suggest a different and more convincing explanation.

After 3 days of the GCED, all urinary samples were indeed GIP-negative, whereas a challenge with minute amounts of gluten (5 or 10 mg) was sufficient to cause positivity of urinary GIP in 33% of cases. In other words, a true zero-gluten diet (the GCED) was constantly associated with a negative urinary GIP test, whereas the traces of gluten that may be found in commercially available gluten-free food (that may generate an intake of up to 10 mg/d of gluten by definition) yielded a positive result in a significant proportion of cases. Therefore, the urinary GIP test seems to be somewhat too sensitive and may result positive even in subjects perfectly complying with the requirements of the standard GFD. These findings have an important clinical implication. The previously reported high rate of positive GIP tests in patients with CD on a GFD should not be interpreted as evidence of poor compliance to the GFD, an issue that has raised many concerns in the real life of celiac patients. On the other hand, this procedure might find application in the monitoring of hypersensitive patients with CD treated by the GCED.

Our study disclosed further limitations of the urinary GIP test, first the high percentage (25%) of negative urinary GIP results after macrodoses of gluten (500–1,000 mg). Whether the consequence of the complete digestion of GIP into the gut, for example, related to a specific proteolytic activity of the intestinal microbiota (9), or whether caused by some other unknown factor, for example, an abnormal intestinal transit time, this result indicates that the negative predictive value of the test is poor (Figure 5). Another still unclear issue is the kinetics of urinary GIP elimination. It was originally suggested that GIP are detectable in urine only between 3 and 9 hours from gluten ingestion. However, we found a similar number of postgluten challenge urinary GIP positives in the T_0_–T_9_ and the T_10_–T_24_ urine collections, suggesting that delayed GIP elimination is common. Finally, we did not find any dose/effect relationship between the quantity of ingested gluten and the amount of urinary GIP (Figure 3). All these findings suggest that the urinary GIP test may not be an effective tool for monitoring a GFD adherence and seriously dispute the validity of studies estimating the amount of contaminating gluten in a diet through the application of a complex (and largely theoretical) conversion factor to the concentration of GIP in a random urine sample (15). On the other hand, the diagnostic accuracy of stool GIP determination remains to be evaluated.

The strengths of this study are the accuracy of the study design, the reliability of participants, the standardization of the gluten challenge, and the large number of challenge procedures. The weaknesses are the small size of the study group, the impossibility to fully control the complete adherence to the GFD in a real-life scenario, and the use of raw (rather than cooked) gluten for the challenge procedures. It also remains to be clarified whether our findings may extend to patients with CD, although the standardization of the urinary GIP test in patients frequently showing a variable degree of intestinal mucosa damage, as it is the case in subjects with treated CD (25), could prove even more difficult than in healthy controls.

In conclusion, this study suggests that the urinary GIP determination may not be an accurate method to assess the adherence to the standard GFD, but the test may find application in monitoring a zero-gluten diet as the GCED. Additional validation studies are needed to investigate the diagnostic accuracy and the dose/response of GIP determination in stool.

## CONFLICTS OF INTEREST

**Guarantor of the article:** Carlo Catassi, MD, MPH, is the guarantor of the article and fully responsible for the conduct of the study.

**Specific author contributions:** C. M., A.K.V.: investigation, data curation, writing-original draft, writing-review and editing; G.N.C.: investigation, writing-review and editing; E.F., S.G.: conceptualization and methodology, writing-review and editing; R.G.: software and formal analysis; E. L., C.C.: conceptualization and methodology; writing-review and editing; C.C.: visualization and supervision; project administration. All authors read and approved the final version of the manuscript.

**Financial support**: None to report.

**Potential competing interests**: C.C. is a scientific consultant to Dr. Schär Food, Takeda, and NOOS Italy. The other authors declare no conflicts of interest.

**Ethical clearance**: The study was conducted in accordance with the principles of the Helsinki Declaration as revised in Fortaleza 2013 and was approved by the Ethical Committee of the Polytechnic University of Marche, Ancona, Italy (ID # 131530). The trial was registered in the clinicaltrials.gov registry (ClinicalTrials.gov ID # NCT04477239).

**Study Highlights**

**WHAT IS KNOWN**

✓ The evaluation of adherence to the gluten-free diet is an unmet need in celiac patients.
✓ Growing interest has focused on the determination of stool/urinary gluten immunogenic peptides (GIP).

**WHAT IS NEW HERE**

✓ Urinary GIP are frequently positive in subjects adhering to the gluten-free diet.
✓ Urinary GIP remain negative in subjects ingesting significant amounts of gluten.

## Supplementary Material

SUPPLEMENTARY MATERIAL
