# Supplementary material for: Determination of Urinary Gluten Immunogenic Peptides to Assess Adherence to the Gluten-Free Diet: A Randomized, Double-Blind, Controlled Study
Source: Clin Transl Gastroenterol. 2021 Oct 6;12(10):e00411. doi: 10.14309/ctg.0000000000000411 (PMC8500619; doi:10.14309/ctg.0000000000000411)
Supplement: SUPPLEMENTARY MATERIAL [file ct9-12-e00411-s001.docx]

**Gluten-Free Diet (GFD) protocol**

| **Naturally gluten-free foods*** | **Certified**  **gluten-free foods** | **Prohibited foods** |
| --- | --- | --- |
| All types of meat not mixed with other ingredients | Bread, Pasta, biscuits, cookies, cereal mix | Wheat, emmer, einkorn, spelt |
| All types of fish, shellfish and crustaceans (fresh or frozen) not mixed with other ingredients | Flours (rice, corn, chestnuts, chickpeas, soy, almonds, hazelnuts) | Oats |
| Eggs | Lentils, buckwheat, amaranth, millet, quinoa | Barley |
| All legumes as they are (beans, peas, chickpeas, lupins, broad beans, soy) | Cured meats and sausages made from pork, beef or poultry | Rye |
| Rice (kernels) | Meat preserves (e.g. canned meat, in jelly) | Meat or fish breaded or mixed with breadcrumbs (hamburgers, meatballs, etc.) or cooked in gravies and sauces thickened with prohibited flours |
| Corn (kernels, steamed) | Preserved fish (natural, in oil, smoked) | Barley and/or wheat malt beer |
| Vegetables (fresh, dried, frozen, deep-frozen) | Ready risottos (in containers, frozen, flavored) | Chocolate with prohibited grains |
| Fresh fruits | Labeled gluten-free beer | Yeast base or sour yeast |
| Potatoes | Crackers, breadsticks, taralli, croutons | Seitan |
| Milk (without added vitamins, flavorings or other substances), white yogurt (without added flavorings or other substances) and natural yogurt | Cream | Couscous |
| Fresh and aged cheeses (with safe ingredients) | Sliced, melted, light, spreadable, vegetable cheeses | Kamut |
| Coffee | Pre-fried, pre-cooked frozen potatoes | Floured dehydrated fruit (e.g. dried figs) |
| Tea (decaffeinated), chamomile (filtered) | Instant or frozen puree | All foods containing malt or malt extract of prohibited cereals |
| Wine (traditional, sparkling) | Packaged potato chips (snacks) | Supplements |
| Oil | Preparations for minestrone consisting of vegetables and other ingredients |  |
| Butter | Preserved, steamed/boiled vegetables mixed with other ingredients |  |
| Parmigiano Reggiano DOP and Grana Padano DOP cheeses | Grilled vegetables (in brine, in oil, frozen) |  |
| Honey, sugar (white, cane, granulated) | Melba toast |  |
| Pepper, salt, saffron, spices and aromatic herbs | Packaged sweet snacks |  |
|  | Creams, puddings, desserts |  |
|  | Ice-creams |  |
|  | Chocolate |  |
|  | Dehydrated fruit, candied, caramelized, glazed fruit |  |
|  | Smoothie |  |
|  | Light drinks, "zero", with added sweeteners |  |
|  | Fruit drinks |  |
|  | Tea, chamomile, herbal teas (liquid, soluble and powdered preparations) |  |
|  | Bouillon cube, prepared broth |  |

*Please look carefully product’s ingredient for any gluten-containing agent

*Do not buy any products with label may contain gluten traces
