# Supplementary material for: Determination of Urinary Gluten Immunogenic Peptides to Assess Adherence to the Gluten-Free Diet: A Randomized, Double-Blind, Controlled Study
Source: Clin Transl Gastroenterol. 2021 Oct 6;12(10):e00411. doi: 10.14309/ctg.0000000000000411 (PMC8500619; doi:10.14309/ctg.0000000000000411)
Supplement: SUPPLEMENTARY MATERIAL [file ct9-12-e00411-s002.docx]

**Gluten Contamination Elimination Diet (GCED)**

**Allowed food:**

- Fresh meat (unprocessed)
- Fresh fish
- Fresh vegetables
- Fresh legumes
- Fresh fruit
- White yogurt (without additives)
- Eggs
- Milk
- Potatoes
- Rice in grains
- Dried fruit (unshelled)
- Coffee (moka pot), tea, herbal teas
- Oil
- Salt
- Sugar

(NO medications or supplements)
